# Simulation of thermal stress and buckling instability in Si/Ge and Ge/Si core/shell nanowires

**DOI:** 10.3762/bjnano.6.201

**Published:** 2015-10-02

**Authors:** Suvankar Das, Amitava Moitra, Mishreyee Bhattacharya, Amlan Dutta

**Affiliations:** 1 S. N. Bose National Centre for Basic Sciences, Sector - III, Salt Lake, Kolkata 700098, India; 2 Variable Energy Cyclotron Centre, 1/AF, Bidhannagar, Salt Lake, Kolkata 700064, India

**Keywords:** atomistic simulation, buckling, core–shell nanowire, thermal stress

## Abstract

The present study employs the method of atomistic simulation to estimate the thermal stress experienced by Si/Ge and Ge/Si, ultrathin, core/shell nanowires with fixed ends. The underlying technique involves the computation of Young’s modulus and the linear coefficient of thermal expansion through separate simulations. These two material parameters are combined to obtain the thermal stress on the nanowires. In addition, the thermally induced stress is perceived in the context of buckling instability. The analysis provides a trade-off between the geometrical and operational parameters of the nanostructures. The proposed methodology can be extended to other materials and structures and helps with the prediction of the conditions under which a nanowire-based device might possibly fail due to elastic instability.

## Introduction

In recent years, a drastic rise in the research activities on semiconductor core/shell nanowires (CSNWs) made of silicon and germanium has occurred. Such studies are often motivated by the excellent charge transport properties of the materials [1–4], for which they are now seen as prospective candidates for next generation transistor devices. The radial heterostructure offers the advantage of control of the band gap and charge carrier mobility by tuning their size [5] and selecting suitable impurity doping scheme [3,6]. In addition, they exhibit significantly suppressed phonon thermal conductivity and suitability as novel thermoelectric devices [7]. This effect is found to be extremely prominent for ultrathin nanowires with diameters smaller than ≈20 nm [8]. The figure of merit can be further improved by introducing surface disorder without compromising the excellent electronic conductance [9].

In many experimental studies, the heterostructured nanowires are allowed to grow freely though physical or chemical routes [3,6]. Nevertheless, as components of nanoelectronic or nanoelectromechanical systems, the wire is expected to have fixed boundaries. In all such scenarios, the temperature becomes a crucial operating parameter for the CSNW devices. On one hand, it directly affects the transport properties through phononic contributions, while on the other, it can induce thermal stress in the system. Although the former effect can be quantified through direct transport measurements [2,10], it is prohibitively difficult to experimentally measure the thermal load on ultrathin CSNWs. The effect of thermal stress on the performance of the device is again two-fold. The mechanical load would alter its electronic band structure and charge carrier mobility [11–13], which is particularly significant on account of the large piezo resistance at the nanoscale [14]. In addition, the axial compressive stress at elevated temperature can cause elastic instability and buckling of a slender one-dimensional nanostructure [15], thereby jeopardizing the structural integrity of the system. This is evidently a matter of concern as various types of operating conditions can expose the nanowires to a broad range of temperatures. Clearly, the issue of thermal stress requires careful consideration before designing the CSNW-based devices for practical applications.

This work attempts to quantify the axial normal stresses on clamped Si/Ge and Ge/Si CSNWs due to variation in the operating temperature. The calculation of thermal stress typically involves the measurement of Young’s modulus and the thermal expansion of a solid. Unlike the composite structures of macroscopic dimensions, core–shell nanowires present features that restrict the prediction of the overall elastic modulus through simple analytical means. Decomposing the system into an isolated nanowire (core) and outer tube (shell) to apply the parallel spring model yields incorrect results due to the effect of the core–shell interface [16]. Moreover, the mismatch between the lattice parameters of Si and Ge causes an inhomogeneous strain distribution within the unloaded CSNW [17–18]. The strain field is stronger near the core–shell interface and may induce some elastic nonlinearity, thereby dictating the overall elastic moduli of the composite system. In the same way, the presence of an interface modulates the state of phonons in these nanowires and the analytical estimation of the net thermal expansion by conventional thermo-mechanical theory becomes unfeasible. In view of these difficulties, atomistic simulations have been employed to obtain the elastic constants and coefficients of thermal expansion (CTE) of these semiconductor heterostructures. The coupling of these two parameters yields a reasonable estimate of the magnitude of thermal stress endured by the system. Once the thermal stress is obtained, it is compared to the critical stress for elastic instability of the nanowire, which furnishes the safe limits of designing and operating parameters of these nanowire devices.

## Results and Discussion

### Simulation scheme

The present study involves a series of molecular statics (MS) and molecular dynamics (MD) simulations of core/shell nanowires. As we focus on the applications of 1D devices with ultrasmall lateral dimensions (≈10 nm diameter), we can assume the CSNW to have a coherent structure across the core/shell interface. This assumption is reasonable, for the propensity of epitaxial semiconductor heterostructues to accommodate misfit dislocations is significant only at larger diameters [1,19–20]. For constructing the core/shell wires, a strategy similar to that used by Liu et al. [16] has been employed. Although their analyses were for the wires of hexagonal cross-sections, the present investigations are based on the cylindrical CSNWs with circular cross-sections. This is because although GaAs/GaAlAs nanowires with core/shell morphology can form hexagonal cross-sections [21], their Si–Ge-based counterparts are typically depicted as having circular cross-sections in several experimental [3,8,22] and theoretical studies [9,23–24]. The simulated CSNWs have a single crystalline, pseudomorphic diamond-cubic configuration and the effective lattice constant is obtained by gradually varying the lattice constant of the system within a range [16]. The lattice constant yielding the minimum energy (per atom) after structural relaxation is considered as the optimized equilibrium parameter for a given core/shell configuration. Experiments have suggested these CSNWs to have a sharply distinct interface with negligible interdiffusion of atoms [25]. Even if some intermixing is possible, it can occur only if the NW is annealed at a very high temperature for a long duration. Usually, the interdiffusion decreases the interfacial stress. As the Si–Ge lattice mismatch is only about 4% and the CTEs are measured at room temperature, a clean and sharp interface is chosen, which has also been preferred in other simulation studies [7,9,16]. We simulate both Si/Ge and Ge/Si CSNWs oriented along the <111> and <110> directions. For each case, wires with an outer diameter of 10 nm are created, while the core diameter is varied to generate a series of structures. The simulation cell is periodic along the axial diameter, so that an infinitely long, freestanding NW is effectively simulated. In these simulations, the Tersoff potential designed for multicomponent systems [26] has been used to model the interatomic interactions. Although this form of potential does not capture the features of surface reconstruction very well, it would be significant only for a nanowire diameter of less than 2–3 nm. As the surface/volume ratio decays rapidly with increasing wire thickness, the surface reconstruction is not expected to dramatically affect the Young’s modulus or thermal expansion coefficient of 10 nm diameter nanowires.

**Figure 1:**
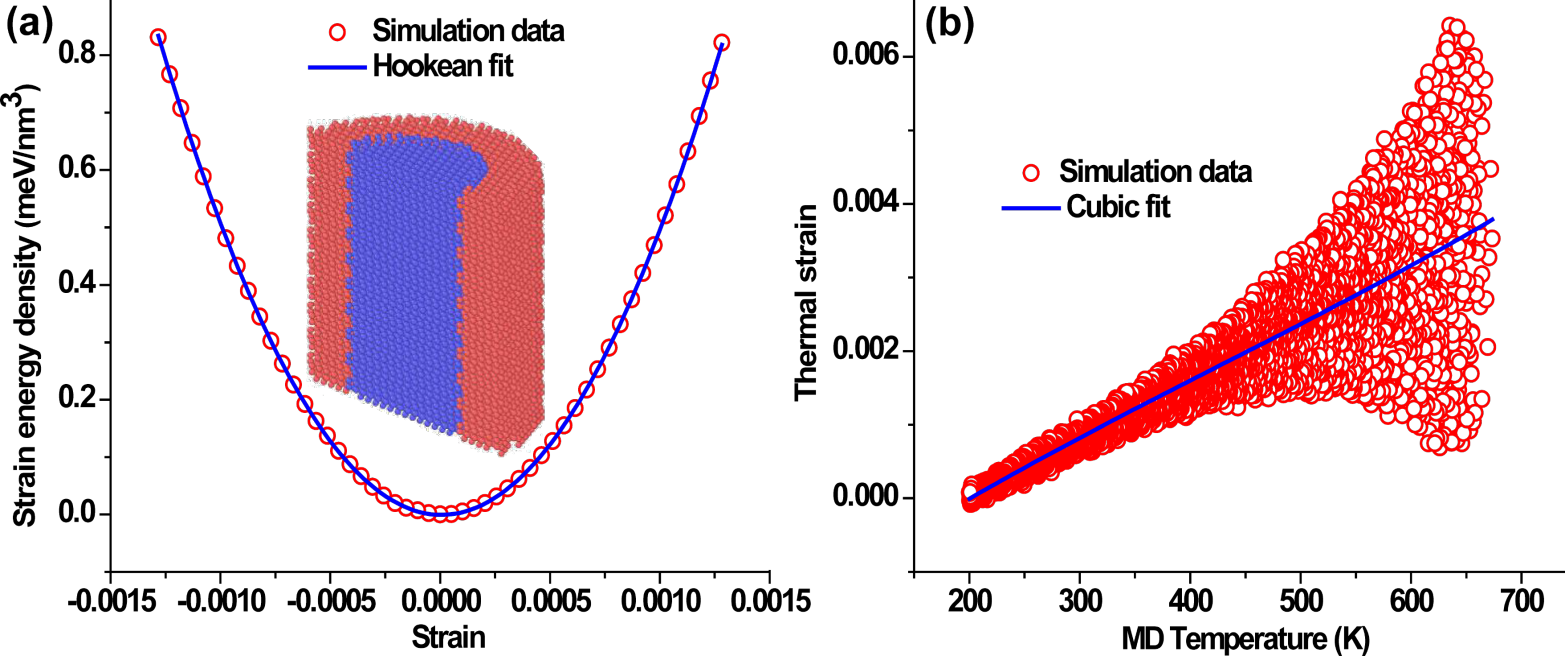
Methods for computing Young’s modulus and the thermal expansion coefficient: (a) Young’s modulus is computed by obtaining the strain energy as a function of longitudinal strain. The cross-sectional view of a typical CSNW is also shown. (b) The thermal expansion is obtained from the thermal strain in a direct MD simulation. At higher temperatures, the thermal fluctuations increase, thereby causing the MD data to be dispersed more broadly. The plots presented here correspond to a Si/Ge, <111> CSNW with a 4 nm core diameter.

The computation of the Young’s modulus (*E*) of the composite system is done in a straightforward manner by imposing longitudinal strain (ε) in small increments along the axial direction. Each step of strain increment is followed by structural relaxation and the effective modulus is obtained by fitting the parabolic Hookean strain energy density, *U* = *E*ε^2^/2, to the energies obtained from the simulations (Figure 1a). For observing the thermal expansion of the CSNW, the system is thermally equilibrated at 650 K and cooled to 200 K at 0.75 K/ps, while the normal stress on the wire approaches zero (NPT ensemble). The length of the simulation cell in the axial direction fluctuates during the MD simulation (Figure 1b). A derivative of the third-order polynomial fit to the thermal strain vs temperature results is used to obtain the coefficient of thermal expansion, α. The open source MD codes LAMMPS [27] and MD++ [28] have been employed for performing the simulations. Wherever required, the crystal structure was viewed using the OVITO [29] visualization tool.

### Measurement of thermal stress

**Figure 2:**
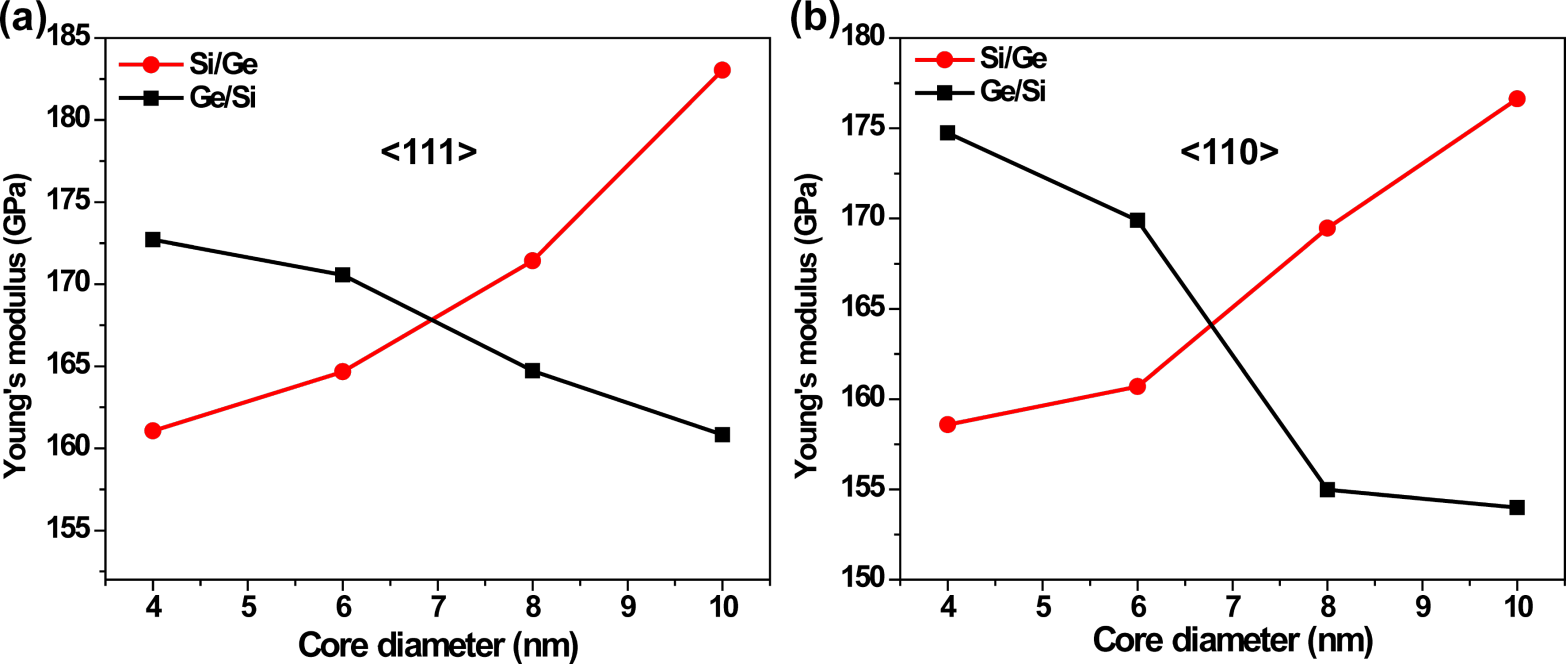
Variation of Young’s modulus with the core diameter of the (a) <111> and (b) <110>-oriented core/shell nanowires. The Si/Ge and Ge/Si structures are found to exhibit opposite trends. The largest core diameter of 10 nm corresponds to the pure, monoelemental nanowires.

As mentioned in the previous section, CSNWs with different core diameters have been simulated for a fixed outer diameter of 10 nm. Figure 2a,b presents the variation of Young’s modulus for the nanowires of different core diameters. For both <111> and <110> orientations, the Young’s modulus of the Si/Ge wire increases monotonically with an increasing core diameter. In contrast, the Ge/Si wire exhibits the opposite trend. This behavior can be understood simply by observing the elastic modulus of the monoelemental NWs (represented as 10 nm core diameter). Silicon, being stiffer than germanium, shows a general tendency of an increasing Young’s modulus with a rise in its compositional contribution in the hetrerostructure. Interestingly, the crossover between the elastic modulus of Si/Ge and Ge/Si NWs occurs near a core diameter of ≈7 nm. This is a structure with equal volume occupied by the core and shell parts of the material.

**Figure 3:**
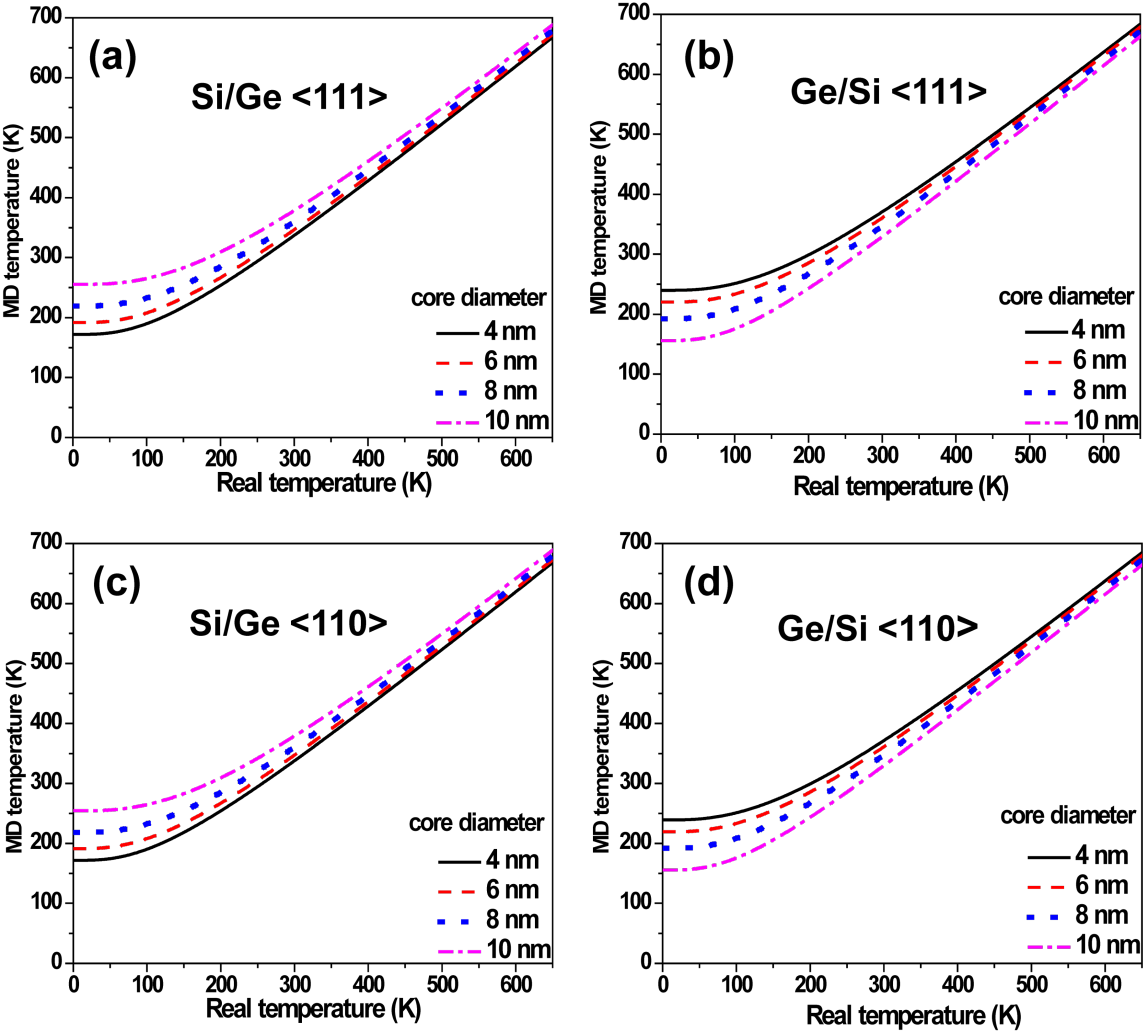
Quantum scaling between the real and classical (MD) temperatures for the (a) Si/Ge <111>, (b) Ge/Si <111>, (c) Si/Ge <110> and (d) Ge/Si <110> CSNWs. The minimum nonzero MD temperature corresponding to 0 K (real temperature) reflects the zero-point vibrations of the atoms.

The next step of our computation involved the estimation of coefficient of thermal expansion (CTE), using the methodology outlined in the previous section. The linear CTE is defined as,

[1]
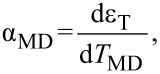


where the temperature derivative of the thermal strain (ε_T_) is given in terms of the classical temperature, *T*_MD_. For solids with a large Debye temperature, the quantum mechanical effect responsible for the zero-point motion makes the real temperature, *T*, different from the classical temperature, *T*_MD_, as measured from the simulations. Wang et al. [30] proposed an approximate relation for scaling between the classical and real temperatures based on the phonon frequencies in the solid. Accordingly, this procedure has been widely adopted for incorporating the quantum effects in classical MD simulations dealing with the measurement of thermal properties such as thermal conductivity [31] and the coefficient of thermal expansion [32–33]. In this method, a system with *N* atoms produces the 3*N* × 3*N* dynamical matrix, 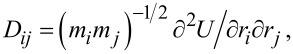 as the second derivative of structural energy with respect to the positional degrees of freedom. In this relation, *m_i_* and *m_j_* denote the atomic mass corresponding to the coordinate variables *r_i_* and *r_j_*, respectively. Assuming the validity of the harmonic approximation model [34], the diagonalization of the dynamical matrix yields the squared-eigenfrequencies (ω^2^) that link the classical and real temperatures of an *N*-atom system:

[2]
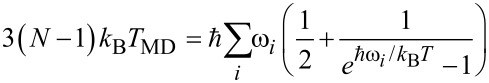


where the symbols have their usual standard meanings. Figure 3a–d displays the classical MD temperatures plotted against their real, quantum mechanical values for all the CSNWs investigated here. The MD temperatures are found to have lower bounds corresponding to the quantum mechanical zero-point energies of the atoms. It can be seen that for both <110>- and <111>-oriented structures, the Si/Ge NWs exhibit a larger zero-point classical temperature with an increase in the core diameter. As the MD temperature always converges towards the real temperature, a larger zero-point temperature entails a smaller slope in the MD vs real temperature plot. In contrast, for the Ge/Si CSNWs, an increase in the core diameter reduces the zero-point MD temperature. Accordingly, the NW with a thicker core corresponds to a larger slope. The measurement of this slope is significant for the real CTE can be scaled to that obtained in Equation 1 as

[3]
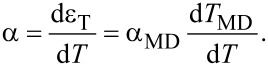


**Figure 4:**
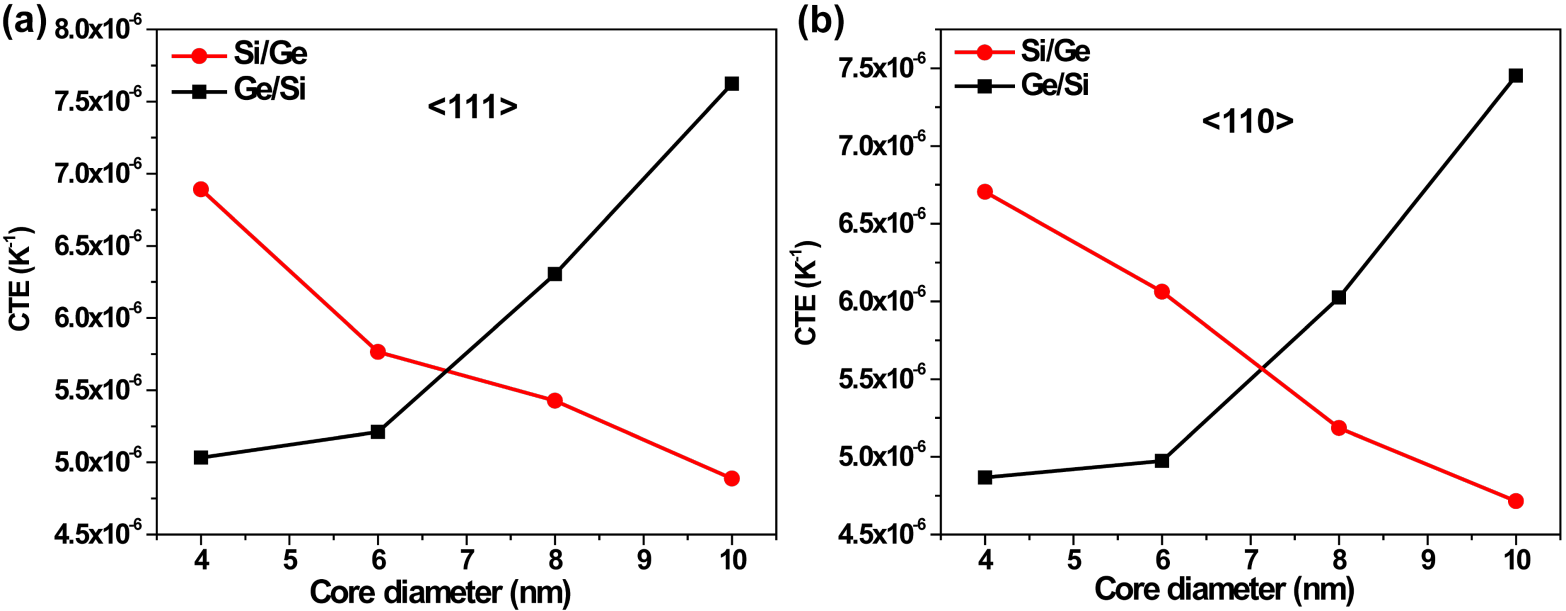
Coefficients of thermal expansion plotted against the core diameter of Si/Ge and Ge/Si CSNWs oriented in (a) <111> and (b) <110> axial directions.

**Figure 5:**
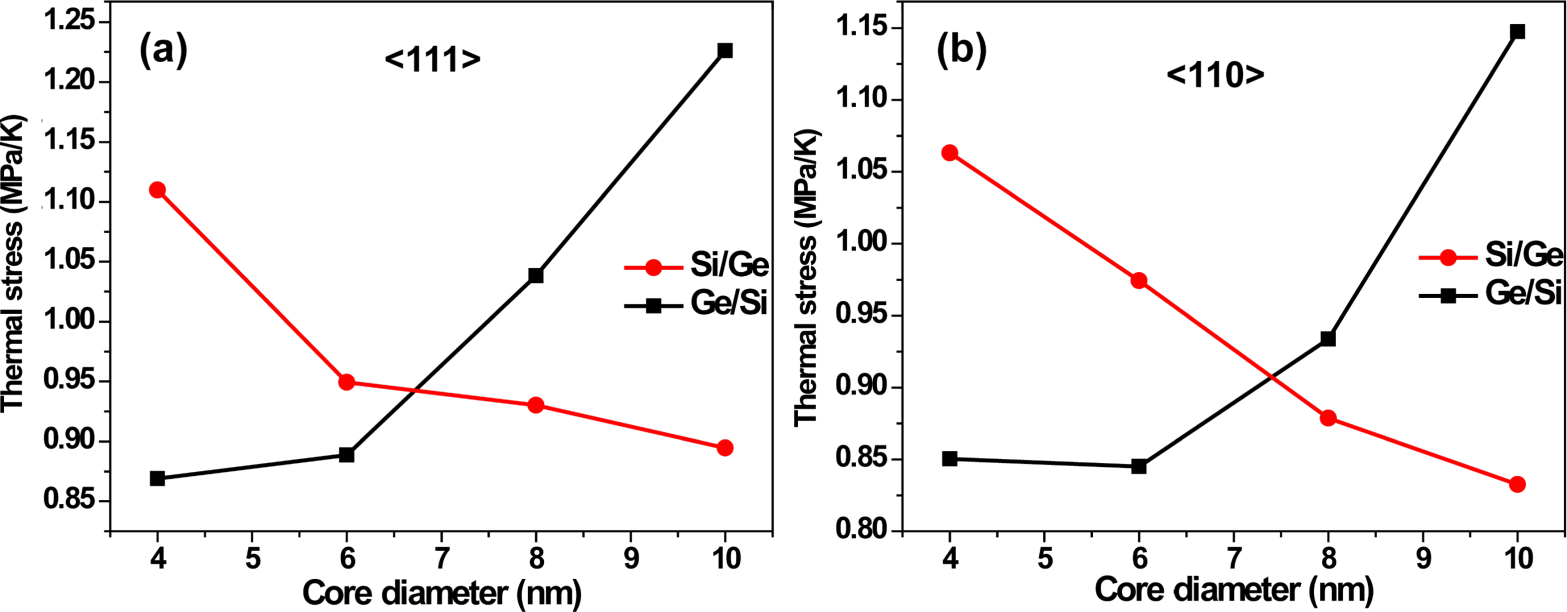
Temperature derivatives of thermal stress at 300 K for the (a) <111>- and (b) <110>-oriented wires of different core diameters.

The above scaling provides the real CTE with quantum mechanical adjustments and are displayed in Figure 4 for *T* = 300 K. We find that the CTE reduces gradually with an increase in the core diameter of the Si/Ge nanowire, whereas the opposite behavior is seen for its Ge/Si counterpart. The variation in CTE with respect to the core diameter is about 50% for the Si/Ge system, and is somewhat narrower for the Ge/Si CSNW. Having obtained the estimate of the thermal expansion, the thermally induced axial stress (σ_T_) on a doubly clamped NW is calculated as dσ_T_/d*T* = α*E*. This value is plotted in Figure 5 and exhibits the same qualitative trend as that of the CTE (Figure 4). This is on account of the fact that a relative change in Young’s modulus with variation in the core diameter is small in both Si/Ge and Ge/Si NWs. Hence, the trend of thermal stress is primarily dominated by the CTE.

### Buckling instability in CSNWs

Elastic instability in a slender solid structure leads to buckling, and is always a major concern in engineering design. When such a structure is under compressive axial load, it can remain stable so long as the stress remains within a critical value. As the applied stress exceeds this upper bound, the elastic equation yields a bifurcated solution and even infinitesimally small perturbation can cause buckling of the structure [35]. Besides significant structures such as machines and buildings, this effect is equally important in the context of nanoscale systems. For instance, this effect is considered while designing loading experiments with nanowires [14]. Recently, the buckling instability has also been associated with the motion of uni-flagellated bacteria [36].

Given that a nanowire, fully clamped at both of its ends, experiences a compressive stress due to a rise in temperature, we can also associate it with buckling instability [15]. A rod of circular cross-section with diameter, *D,* length, *l*, and Young’s modulus, *E*, buckles when the axial compressive stress exceeds the critical value,

[4]
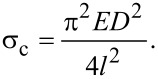


If the rise in temperature has an upper bound, Δ*T*_c_, the critical stress in Equation 4 can be considered as the maximum thermal stress on a doubly clamped NW,

[5]
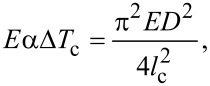


where the critical value, *l*_c_, indicates the maximum allowed wire length corresponding to Δ*T*_c_. Equation 5 can be rewritten as,

[6]
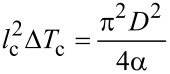


**Figure 6:**
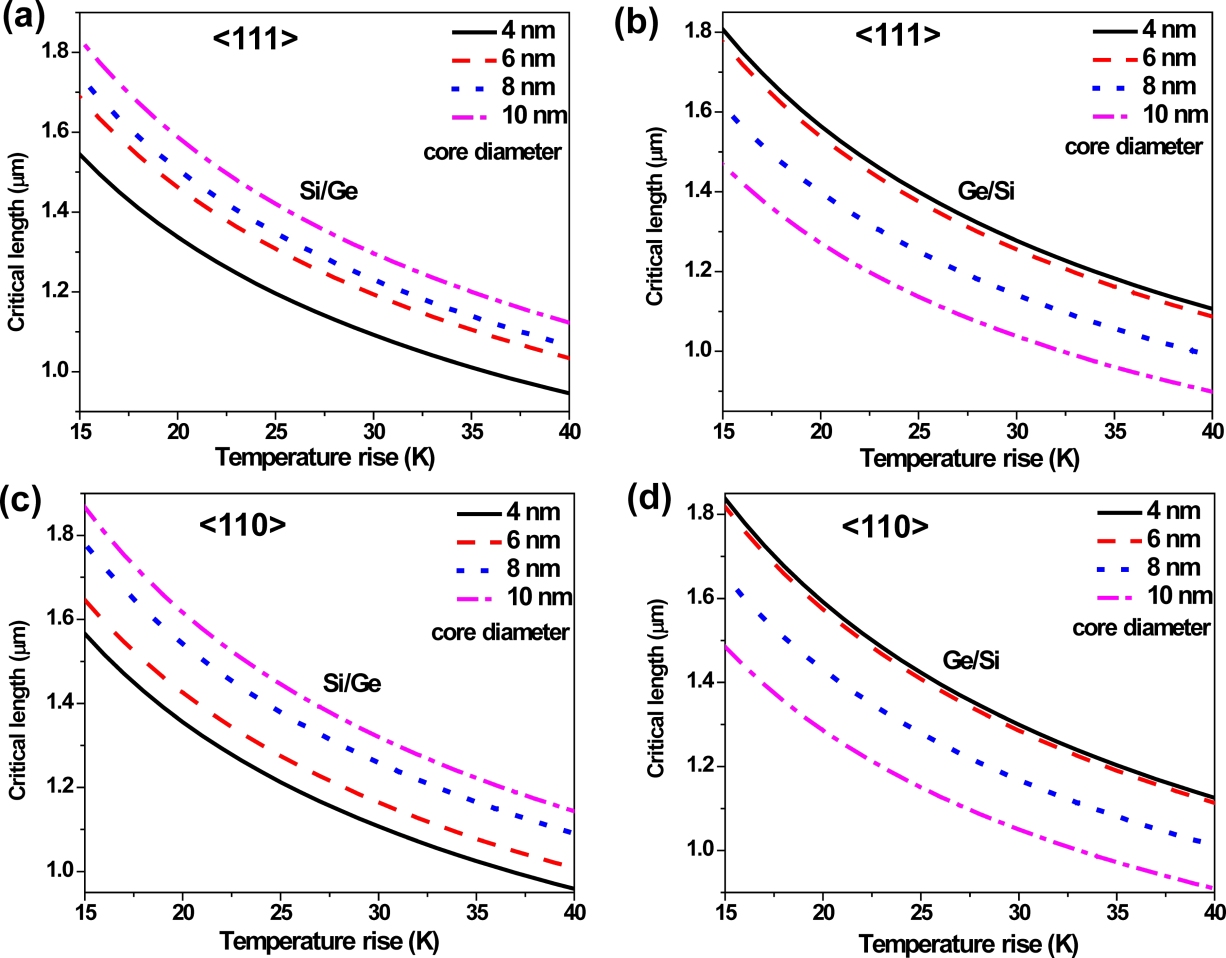
Stability diagrams for CSNWs of (a) Si/Ge <111>, (b) Ge/Si <111>, (c) Si/Ge <110> and (d) Ge/Si <110> configurations. Any combination of temperature increase and nanowire length is safe so long as it remains below the corresponding stability curve.

The above expression is of particular significance as it is an equation-of-state incorporating a geometrical parameter, *l*_c_ (or more generally, the critical aspect ratio (*l*/*D*)_c_) and an operational parameter, Δ*T*_c_. Interestingly, it represents an explicit dependence on the CTE only, which for a composite system like CSNW, can itself depend upon the elastic constants. Equation 6 physically implies a tradeoff between the maximum possible length of the nanowire and the range of its operating temperature. If the nanodevice is to be used under high temperature conditions, its length has to be kept short and vice versa. This is best represented in terms of the stability diagrams shown in Figure 6, where the region below a curve can be considered as stable in terms of wire length and operating temperature range. It can be seen that an elevation in temperature by a few tens of Kelvin can impose severe restrictions on the maximum length of the CSNWs. These plots, which demarcate the regions of stability and instability, are clearly sensitive to the configuration of the CSNWs. In Si/Ge wires, and increase in the core diameter enhances the region of stability for both <110> and <111> orientations. In contrast, a larger core contracts the stability zone for the Ge/Si nanowires.

At this point, it is worthwhile to point out that the above treatment is a first order approximation and there is opportunity for further modifications of a few aspects. First of all, Equation 6 treats the CTE as constant over the entire range of operating temperatures. This may be a reasonable assumption when Δ*T*_c_ remains within a few tens of Kelvin. However, if the temperature varies over a much wider range, or the CTE is highly sensitive to temperature, the temperature dependence, α(*T*), can be considered. This would modify Equation 6 to

[7]
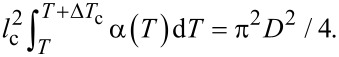


Moreover, the composite elastic constant of the CSNW has been considered in Equation 4 and Equation 5. This is again reasonable in the present case, for the Young's modulus of Si does not differ significantly from that of Ge. It should also be noted that the Tersoff potential overestimates the CTE of silicon [33] and therefore, the extent of the stable zone is somewhat underestimated. Unfortunately a better potential model capable of perfectly reproducing the thermal behavior of the Si–Ge system is yet to be developed. Nevertheless, an overestimation of the CTE still serves the intended purpose, which would not be the case for an underestimated value. This is because in accordance with Equation 6, an overestimated CTE will result in an underestimation of the safe or stable zone in Figure 6. As an underestimated safe zone is a subset of the actual safe region, it will never cover unsafe parameters. Although an exact quantitative prediction is possible if better interatomic potentials are available, Figure 6 still remains useful from an engineering standpoint. This is because for most of the practical applications, where structural stability is important, it is customary to provide a large margin of tolerance to the safe limits of design and operational parameters. This margin is also important in taking care of some other stray effects, such as the thermal expansion of the substrate and clamps. In general, the contributions from such extrinsic factors depend not only on the choice of material, but the design and geometry of the entire setup as well. As a matter of fact, several methods are available to minimize the effect of thermal mismatch between the substrate and components [37]. The methods and results presented in this study can help in making a judicious decision in this regard. In the same way, it is also possible to include the surface effects on the nanostructures [15,38–40], which can slightly modify the end results. Although such refinements can be incorporated in academic interests, the approach of approximate estimation is sufficient for most applications as it is a standard engineering practice to keep the design and operational parameters well below the theoretically permitted maxima.

## Conclusion

In summary, we estimate the thermally induced, axial normal stress on Si/Ge and Ge/Si core/shell nanowires with clamped ends. The underlying method involves the computation of the Young's modulus of the nanostructures through molecular statics simulations. This is followed by molecular dynamics computations, which yield the coefficient of thermal expansion of the materials. Based upon these two physical parameters, the thermal stress has been estimated and was further compared to the critical value corresponding to the buckling instability. Thus, we are able to estimate the safe zone of the NW geometry and operational temperature, where the NW-based device can operate without the risk of elastic structural failure.

Although the present investigation deals with a specific category of a material effect, namely the buckling instability, the proposed computational strategy is a generalized one. With some modifications, it can be employed for other kinds of materials and structures as well. In particular, use of this computational strategy is easier for materials with a lower Debye temperature. Such cases would not require the quantum scaling of temperature, which is the most computationally expensive and prohibitive part of this method for systems with large sizes. Furthermore, some other effects such as the change in transport properties can also be explored, where the thermal stress can play a crucial role. We hope that the present work offers an important guideline for designing practical devices with ultrathin NWs as their vital components.
